# Possible Commonalities of Clinical Manifestations Between Dystonia and Catatonia

**DOI:** 10.3389/fpsyt.2022.876678

**Published:** 2022-04-29

**Authors:** Kanako Ishizuka, Masako Tachibana, Toshiya Inada

**Affiliations:** ^1^Health Support Center, Nagoya Institute of Technology, Nagoya, Japan; ^2^Department of Psychiatry, Nagoya University Hospital, Nagoya, Japan; ^3^Department of Psychiatry, Nagoya University Graduate School of Medicine, Nagoya, Japan

**Keywords:** catatonia, dystonia, biological-genetic overlap, neurodevelopmental disorders, schizophrenia, antipsychotics

In 1874, Kahlbaum described catatonia as a psychomotor syndrome including mutism, negativism, stupor, stereotypies, catalepsy (waxy flexibility), and verbigeration. Kraepelin classified this condition as a subtype of dementia praecox. Bleuler also regarded it as a manifestation appearing in schizophrenia, although he described that it may also appear in other types of illness (1). The current DSM-5 diagnosis of catatonia is diagnosed as signs and postures that display apparent unresponsiveness to external stimuli, despite awakeness. Dystonia is a movement disorder characterized by sustained or intermittent muscle contractions causing abnormal, often repetitive, movements, postures, or both (2). The term is used to describe an uncontrollable muscle spasm that becomes evident as a contraction of the flexor and extensor muscles, leading to an abnormal position. Drug-induced dystonia, which occurs in patients receiving antipsychotic agents, especially schizophrenic patients, appears mostly in the acute phase of antipsychotic initiation. It has been reported to appear in 95% of cases within the first 5 days of starting treatment or increasing the antipsychotic dosage (3). Tardive form of this condition, occurring in patients receiving long-term antipsychotic agents, often shows a gradually progressive fixation of abnormal posture, such as rotation of the necks (torticollis, retrocollis, or anterocollis), oculogyric crisis, and Pisa syndrome (Figure 1), which sometimes requires differentiation from catatonia due to the similarity of clinical manifestations between dystonia and catatonia. If antipsychotic-induced dystonia can be ruled out in diagnosing the differentiation or comorbidity between catatonia and dystonia, the possibility that the catatonia concomitant with comorbid functional (psychogenic) dystonia should also be considered. In fact, the movements of functional dystonia is inconsistent with those seen in other forms of dystonia, and the movements themselves are often internally inconsistent with several different semiologies, including catatonia, may coexist in the abnormal posture (4).

**Figure 1:**
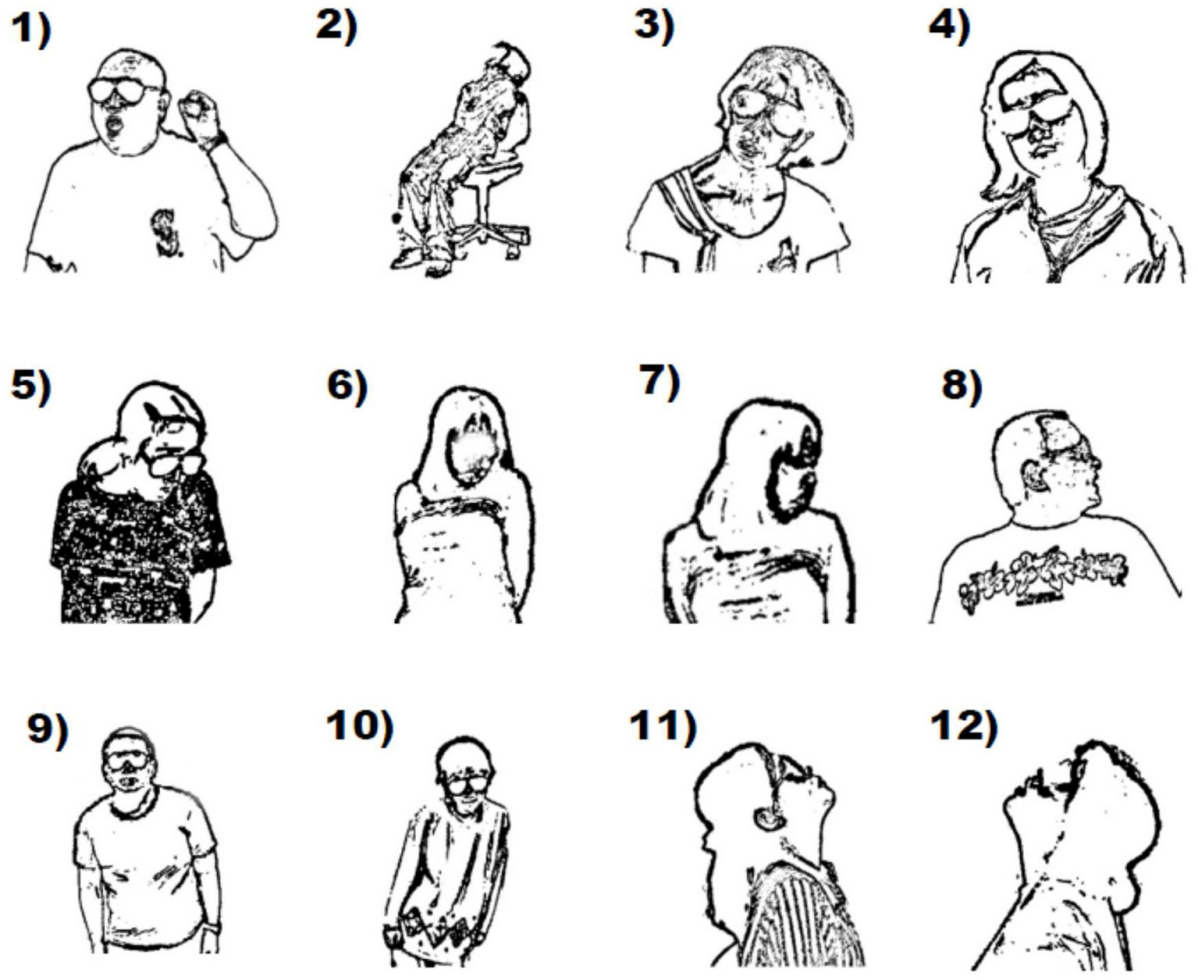
Monochrome images of dystonic abnormal postures extracted from videoclips observed in schizophrenic patients receiving antipsychotics. **(1)** co-occurrence of cervical dystonia (mild torticollis) and catatonic posture (lifting a left arm); **(2)** trunk dystonia; **(3,4)** cervical dystonia (laterocollis); **(5,6)** cervical dystonia (mild torticollis with anterocollis); **(7,8)** cervical dystonia (severe torticollis); **(9,10)** Pisa syndrome; **(11,12)** cervical dystonia (retrocollis).

Although catatonia affects a person's mental functioning and behavior, motor symptoms have been neglected compared to cognitive and affective symptoms. However, emerging theoretical and methodological advancements have shed new light on the utility of considering movement abnormalities leading to the characteristic abnormal posture. The National Institute of Mental Health Research Domain Criteria initiative recently met to develop a Motor Systems Domain to explore and understand different motor functions in the context of psychiatric illnesses, given that motor abnormalities are not epiphenomena of mental illnesses; they appear to be intrinsically linked to disease processes that give rise to the cognitive, emotion regulation, and social deficits that are trademarks of these mental disorders (5, 6). Because movement disorders are complex phenotypes with multiple genes impacting the probability of onset, the differential diagnosis poses great challenges, given the considerable overlap of signs and symptoms between the conditions.

Involvement of the dopaminergic system has been consistently associated with the symptoms of catatonia and dystonia. Antipsychotic agents commonly cause dystonia, supporting a role for the dopaminergic network in its pathophysiology. In addition, recent evidence from genetic studies further corroborates the involvement of striatal dopaminergic dysfunction in the imbalance between the direct and indirect basal ganglia pathways, as well as central involvement of striatal dopamine in dystonia pathogenesis (7). Aberrant thalamo-cortical and cortico-cerebellar circuits modulated by dopaminergic transmission are known as relevant neural circuits underlying catatonia and dystonia. Both movement abnormalities have been convincingly linked to the disease process by their presence in antipsychotic-naïve schizophrenic patients and their first-degree relatives (8). Furthermore, magnetic resonance imaging studies have demonstrated that sensorimotor abnormalities are associated with structural and functional changes within the cortical–thalamic–cerebellar–cortical circuit, which is intricately linked to schizophrenia itself. In line with this, sensorimotor abnormalities appear to be intrinsic to schizophrenia in a manner that can be improved or exacerbated by antipsychotic medication (9).

Historically, most of the literature on motor abnormalities has focused on schizophrenia, so their prevalence and featural properties in other neuropsychiatric disorders are poorly known (10). In this note, we propose the significance of focusing on molecular neurogenetic commonalities of these conditions as a model. Catatonia is a prominent feature in patients with neurogenetic disorders such as autism, Down syndrome, Phelan–McDermid syndrome, Kleine–Levin syndrome, Prader–Willi syndrome, and 22q11.2 deletion syndrome (11–13) and these neurogenetic disorders are well-known among child psychiatrists. More than 10% of adolescents and young adults with autism (14) and 53% of individuals with Phelan–McDermid syndrome (15) experience catatonia. This high comorbidity of catatonia among patients with neurogenetic disorders is not well-recognized among adult psychiatrists, and it may often be regarded as a catatonic subtype of schizophrenia or other functional psychiatric disorders such as mood disorders. The study of dystonias in some neurogenetic diseases such as Down syndrome and 22q11.2 deletion syndrome that present with catatonic features comorbid with dystonia deserves more attention (16, 17). Riccio et al. reported a case of autism presenting among the catatonic symptoms of fixed posture and motor overflow that assumed dystonic characteristics (18). Taken together, both catatonia and dystonia may present with a broad spectrum of motor abnormalities in individuals with neurogenetic disorders.

Because advances in medical science mean it is no longer uncommon for patients with these neurogenetic disorders to survive into adulthood, an urgent need exists to better understand and manage the persisting and later-onset features, including dystonia and catatonia, of the neuropsychiatric manifestations (17). Catatonia has been reclassed from a subtype of schizophrenia to a condition and a specifier that can be observed in any psychiatric or medical disorder, including neurogenetic disorders, in the DSM-5 since 2013. Establishing the possible associations between dystonia and catatonia in the context of neurogenetic disorders without the frame of schizophrenia, along with their similarity in clinical manifestations, may help improve management and allow more accurate and timely diagnosis for these conditions. Prospective longitudinal studies based on standardized assessments for motor symptoms, including careful history-taking, physical examination, and use of validated rating scales, in addition to molecular genetic findings, could improve diagnostic biological markers. The possible biological-genetic overlap among these conditions, as well as the possible neurogenetic commonalities of their similar clinical manifestations, could be elucidated.

## Author Contributions

KI, MT, and TI planned, organized, and selected the article be included in the special issue. All authors contributed to the article and approved the submitted version.

## Funding

This work was partly supported by JSPS KAKENHI Grant Numbers JP17H06747, JP18K15513, JP19K08071, and JP20K16625.

## Conflict of Interest

The authors declare that the research was conducted in the absence of any commercial or financial relationships that could be construed as a potential conflict of interest.

## Publisher's Note

All claims expressed in this article are solely those of the authors and do not necessarily represent those of their affiliated organizations, or those of the publisher, the editors and the reviewers. Any product that may be evaluated in this article, or claim that may be made by its manufacturer, is not guaranteed or endorsed by the publisher.
